# Peste des Petits Ruminants Virus Tissue Tropism and Pathogenesis in Sheep and Goats following Experimental Infection

**DOI:** 10.1371/journal.pone.0087145

**Published:** 2014-01-30

**Authors:** Thang Truong, Hani Boshra, Carissa Embury-Hyatt, Charles Nfon, Volker Gerdts, Suresh Tikoo, Lorne A. Babiuk, Pravesh Kara, Thireshni Chetty, Arshad Mather, David B. Wallace, Shawn Babiuk

**Affiliations:** 1 National Centre for Foreign Animal Disease, Canadian Food Inspection Agency, Winnipeg, MB, Canada; 2 Vaccine and Infectious Disease Organization, University of Saskatchewan, Saskatoon, SK, Canada; 3 School of Public Health, University of Saskatchewan, Saskatoon, SK, Canada; 4 University of Alberta, Edmonton, AB, Canada; 5 ARC-Onderstepoort Veterinary Institute, Onderstepoort, South Africa; 6 Department Veterinary Tropical Diseases, Faculty Veterinary Science, University of Pretoria, Pretoria, South Africa; 7 University of Manitoba, Winnipeg, MB, Canada; University of Liverpool, United Kingdom

## Abstract

Peste des petits ruminants (PPR) is a viral disease which primarily affects small ruminants, causing significant economic losses for the livestock industry in developing countries. It is endemic in Saharan and sub-Saharan Africa, the Middle East and the Indian sub-continent. The primary hosts for peste des petits ruminants virus (PPRV) are goats and sheep; however recent models studying the pathology, disease progression and viremia of PPRV have focused primarily on goat models. This study evaluates the tissue tropism and pathogenesis of PPR following experimental infection of sheep and goats using a quantitative time-course study. Upon infection with a virulent strain of PPRV, both sheep and goats developed clinical signs and lesions typical of PPR, although sheep displayed milder clinical disease compared to goats. Tissue tropism of PPRV was evaluated by real-time RT-PCR and immunohistochemistry. Lymph nodes, lymphoid tissue and digestive tract organs were the predominant sites of virus replication. The results presented in this study provide models for the comparative evaluation of PPRV pathogenesis and tissue tropism in both sheep and goats. These models are suitable for the establishment of experimental parameters necessary for the evaluation of vaccines, as well as further studies into PPRV-host interactions.

## Introduction

(PPR) is a viral disease that primarily affects small ruminants of commercial importance, such as goats and sheep. Although originally characterized in western Africa in the early part of the 20^th^ century [1], PPR has since been confirmed throughout most of the African continent (excluding southern Africa), as well as the Middle East, central Asia and eastern China [2]. A recent study of sheep and goats in Tunisia found peste des petits ruminants virus (PPRV) seroprevalence of nearly 8% [3]. Clinical signs of the disease vary and may include ocular and nasal discharges, fever, tissue necrosis, and in the majority of cases (70–80%) death of small ruminant livestock occurs within 10–12 days post-infection [4]. In the past decade, the FAO singled out PPR as one of the principal diseases when considering policies pertaining to poverty alleviation prompting the development of international measures to contain outbreaks, which are of particular concern to the economic well-being of African livestock farmers [5]. In 2008, the Kenyan government committed more than one-third of its livestock vaccination budget to combating a national outbreak of PPR.

PPRV has been shown to be the largest member of the *Morbillivirus* genus of single-stranded RNA viruses, with a genome size of 15 948 bp [6]; the genome encodes for 6 proteins, including a nucleoprotein (N), a viral RNA-dependent polymerase (L), an RNA-polymerase phosphoprotein co-factor (P), a matrix protein (M), a fusion protein (F) and a hemagglutinin protein (H) [7]. PPRV has been shown to be transmitted primarily through direct contact with infected animals via secretions or feces [8]. Although only one serotype of PPRV is known to exist, phylogenetic studies indicate that PPRV strains can be divided into 4 distinct lineages: isolates from three of these lineages only occur in Africa, while a fourth is found in both Africa and Asia [9].

Despite the ability of PPR outbreaks to cause widespread deaths in livestock, the precise viral-induced pathogenesis is still not fully understood. Although PPRV-infected animals were known to exhibit clinical signs similar to rinderpest [1], it was not until the late 1970’s that PPR pathogenesis was evaluated in the laboratory [10] [11]. One of these early experiments was described by Bundza *et al.* [12], where a PPRV isolate from an outbreak in Yemen (PPRV-Malig strain) was used to infect both sheep and goats in a controlled environment. It was found that, while some of the typical clinical signs associated with PPR were reproduced, half of the infected goats and sheep survived intranasal inoculation with the virus. These results were in contrast to the high rate of mortality observed in the field. However, despite these differences, the histopathology of samples from infected animals was consistent with those found in naturally infected animals. Since then, other attempts to reproduce the pathology associated with PPR have been performed under experimental conditions [13]–[16], however, results have varied considerably. This is likely due to variations in animal species evaluated (sheep versus goats), variations in viral inoculum preparation, titres inoculated, PPRV isolate utilised and route of inoculation.

Previous field studies in India have found that, while PPRV infection was slightly more prevalent in sheep than in goats in the target population of animals over 3 months old, outbreaks in goats tended to be more severe [17]. Further studies indicated that PPR outbreaks were more common in goats [18]. This suggests that goats may be more susceptible to PPRV infection, although definitive data to support this claim remain elusive. Thus, a comparative study of PPR pathogenesis in sheep and goats using an experimental model to evaluate viral replication, tissue tropism, pathogenesis and immunity was undertaken to gain a better understanding of disease progression between the species. Experimental infections for both sheep and goats with the Yemenese Malig strain [12] were performed and the resulting pathogenesis was evaluated using real time RT-PCR and immunohistochemistry. Currently, attenuated strains of PPRV are used to prevent outbreaks of the disease in Africa and in southern Asia; these include the Nigerian 75/1 strain [19], as well as the south Asian isolates Sungri 96, Arasur 87 and Coimbatoire 97 [20]. Furthermore, capripoxvirus vaccine vectors have been shown to elicit immunity against PPRV when recombinantly expressing PPRV antigens, such as the fusion (F) and/or hemagglutinin (H) proteins [21]–[26]. Therefore, with the increase in the number of experimental vaccines available against PPRV, a standardized model of infection is needed for both sheep and goats to evaluate these and future vaccines.

## Materials and Methods

### Peste Des Petits Ruminants Virus

A stock of PPRV Malig (Yemen) was originally obtained from The Pirbright Institute (Pirbright, U.K.). The viral stock was passaged four times in Vero cells (ATCC) in Dulbecco’s Modified Eagle’s Medium (DMEM) supplemented with 10% fetal bovine serum (FBS) (Multicell Media-Gibco-BRL-USA) and 1% Penicillin/Streptomycin solution (Multicell) in a 37°C incubator with 5% CO_2_. After the third passage, transmission electron microscopy (TEM) was performed to confirm that the virus was still displaying the structural integrity typical of members of the family *Paramyxoviridae*. After the fourth passage, 80%–90% of cells exhibited cytopathic effect (CPE) after 17 days. Infected cells and supernatant were harvested and frozen at −70°C for subsequent challenge experiments. The virus stock was titrated using Vero cells and the TCID_50_ values were determined based on the method of Reed and Muench [27].

### Animals and Experimental Infection

Six Boer cross goats and six Rideau Arcott sheep (all 6-months-old) were housed in separate Biosafety Level 3 animal cubicles at the National Centre for Foreign Animal Disease (Winnipeg, Canada), and were fed a complete balanced diet and water *ad libitum*. Animal experimentation was conducted under the approval of the Canadian Science Centre for Human and Animal Health Animal Care Committee, which follows the guidelines of the Canadian Council on Animal Care. Both sheep and goats were previously screened and found negative for PPRV by real time RT-PCR and serology prior to viral infection. Each individual sheep and goat was infected with Malig PPRV (2 ml delivered intranasally and 2 ml by subcutaneous injection, from a virus stock titrated at 10^4.5^ TCID50/ml). All animals were observed daily, with clinical signs recorded throughout the study. Rectal temperatures were measured daily from 1 to 13 days post infection (dpi). Oral and nasal swabs, as well as blood and sera, were collected from sheep and goats 2 days prior to infection, and 2, 4, 6, 8, 11, 13, 15, 18 and 21 dpi.

### Histology and Immunohistochemistry

One sheep and one goat were euthanized and submitted to a necropsy procedure on each of dpi 6, 8, and 11. Tissues were fixed in 10% neutral phosphate-buffered formalin. Sections were stained with haematoxylin and eosin (HE). For immunohistochemistry, paraffin tissue sections were quenched for 10 minutes in aqueous 3% H_2_O_2_, then pretreated with proteinase K for 10 minutes. Primary antibody, a monoclonal antibody raised against PPRV strain Nigeria 75/1 (generously provided by the USDA, Plum Island, USA), was used at a 1∶1000 dilution in 10% normal goat serum and Tris-buffered saline/0.05% Tween 20 (TBST) solution overnight at 4°C. Labelled tissue sections were then stained using a horseradish peroxidase-labelled polymer (Envision®+system [anti-mouse] [Dako, USA]), reacted with the chromogen, diaminobenzidine (DAB). The sections were then counter-stained with Gill’s hematoxylin.

For double-immunostaining, paraffin tissue sections were quenched for 10 minutes in aqueous 3% H_2_O_2_, then pretreated with proteinase K for 10 minutes. The primary antibody was applied and developed as described above. The slides were then incubated for 5 minutes with Biocare denaturing solution (Biocare, USA). The second primary antibody, a mouse monoclonal antibody specific for CD68 (EMB11) (Dako), was used at a 1∶20 dilution in TBST solution overnight at 4°C. Sections were then stained using an alkaline phosphatase-labelled polymer (Mach 4 universal system® [Biocare]), reacted with the chromogen, Vulcan Fast Red (VFR). The sections were then counter-stained with Gill’s hematoxylin.

### Virus Isolation from Sheep and Goat Tissue Samples

Tissues from sheep (Table 1) and goats (Table 2) were homogenized using 2.0 mm Zirconia beads (BioSpec Products, USA), and a 10% homogenate was prepared in DMEM supplemented with 1% Penicillin/Streptomycin solution (Multi Cell, USA), as previously described by Hammouchi *et al.* [14]. 500 µl of supernatant was used to infect Vero cells cultured in 75 cm^2^ flasks grown in a 37°C incubator with 5% CO_2_. Cells were evaluated daily for CPE for 20 days.

**Table 1:** Real-time RT-PCR, H&E and IHC result comparison for sheep euthanized on 6, 8 and 11 days post-infection.

	Sheep DPI6			Sheep DPI8			Sheep DPI11		
Tissues	RNA copy/g	H&E	IHC	RNA copy/g	H&E	IHC	RNA copy/g	H&E	IHC
	(log10)			(log10)			(log10)		
Parotid LN	6.4	Y	++++	5.1	Y	++	3.4	Y	+ wk
Retropharyngeal LN	4.8	Y	++++	3.2	Y	+	3.0	Y	+ wk
Bronchial LN	5.7	Y	+++	4.7	Y	+	3.1	Y	−
Prescapular LN	5.6	Y	+++	4.4	Y	+	3.3	Y	+ wk
Mesenteric LN	5.6	Y	+++	4.5	Y	+++		Y	+ wk
Mandibular LN	5.6	Y	++++	4.5	Y	+	2.9	Y	+ wk
Lung - cranial	6.0	Y	++++	1.6	Y	+	1.9	Y	+ wk
Liver	−	N	−	1.9	Y	++	2.0	N	+ wk
Spleen	4.2	Y	++++	1.6	Y	++	1.9	Y	+ wk
Palentine Tonsil	6.2	Y	++++	3.5	Y	++++	5.2	NS	NS
Skin/Lip	NS	Y	+ wk	3.6	Y	+++	2.8	Y	+ wk
Tongue	−	N	−	3.0	Y	+ wk	−	N	+ wk
Trachea	3.2	Y	+ wk	−	N	+	−	N	−
Abomasum	−	N	−	4.6	N	−	−	N	−
Rumen	−	N	−	3.9	Y	+	4.1	N	+ wk
Reticulum	−	Y	+	−	N	−	5.5	Y	+
Omasum	−	N	−	4.6	Y	+	5.4	N	+ wk
Duodenum	6.0	Y	++++	5.8	Y	++++	−	N	−
Ileum	5.7	Y	++++	7.4	Y	++++	−	Y	++
Caecum	5.9	Y	++++	7.6	Y	++++	−	N	−
Colon	−	Y	+++	7.0	Y	++	−	N	−
Conjunctiva	4.1	Y	++	−	Y	+ wk	−	Y	+ wk
3rd eyelid	5.1	Y	+++	1.7	Y	+	−	Y	+ wk
Pharyngeal mucosa	−	Y	+	−	N	−	−	N	−
Oral mucosa	−	Y	+	NS	Y	+++	−	Y	+ wk
Nasal mucosa	−	Y	+	3.0	Y	+++	−	Y	+

Y = Lesions present consistent with PPR infection.

N = No significant histopathological findings.

NS− No sample.

IHC = Immunohistochemistry score: +wk = weak immunostaining (<20 cells);+ = mild immunostaining (<25% of the section);++ = moderate immunostaining (25% to 50% of the section);+++ = abundant immunostaining (51% to 75% of the section);++++ = extensive immunostaining (>75% of the section).

**Table 2:** Real-time RT-PCR, H&E and IHC result comparison for goats euthanized on 6, 8 and 11 days post-infection.

	Goat DPI6			Goat DPI8			Goat DPI11		
Tissues	RNA copy/g	H&E	IHC	RNA copy/g	H&E	IHC	RNA copy/g	H&E	IHC
	(log10)			(log10)			(log10)		
Parotid LN	3.8	Y	+++	5.1	Y	++	3.0	Y	+ wk
Retropharyngeal LN	4.3	Y	++	5.0	Y	+ wk	3.9	Y	+ wk
Bronchial LN	3.4	Y	++++	4.7	Y	++	3.5	Y	+ wk
Prescapular LN	4.6	Y	++	4.5	Y	++	3.3	Y	+ wk
Mesenteric LN	5.0	Y	++	4.1	Y	+++	4.5	Y	+ wk
Mandibular LN	4.6	Y	++	4.4	Y	++	4.7	Y	+ wk
Lung – cranial	4.5	Y	+	5.2	Y	+++	−	Y	−
Liver	−	N	−	2.1	Y	+ wk	3.1	N	−
Spleen	−	Y	+++	4.0	Y	++	2.8	N	+ wk
Palentine Tonsil	5.5	Y	+++	5.4	Y	++++	3.8	NS	NS
Skin/Lip	3.4	N	+ wk	2.9	Y	+++	−	N	−
Tongue	−	N	−	−	Y	++++	−	N	−
Trachea	3.6	Y	+	5.2	Y	+++	−	Y	−
Abomasum	−	N	+ wk	4.3	Y	++	−	N	−
Rumen	3.6	N	−	−	Y	+ wk	4.8	Y	+ wk
Omasum	−	N	−	−	Y	−	5.3	N	−
Duodenum	6.0	Y	+ wk	3.4	Y	+++	3.0	Y	+ wk
Ileum	6.3	Y	+++	3.9	Y	++++	3.0	Y	+ wk
Caecum	5.5	Y	++	7.4	Y	++++	2.8	Y	−
Colon	5.5	Y	++	5.2	Y	++++	−	Y	−
Conjunctiva	4.4	N	−	5.7	Y	++	−	Y	+ wk
3rd eyelid	4.4	Y	+	3.3	Y	++	−	Y	+ wk
Pharyngeal mucosa	3.6	N	−	4.3	Y	+	−	N	−
Oral mucosa	−	Y	+ wk	7.8	Y	+++	−	N	−
Nasal mucosa	6.2	Y	+ wk	3.8	Y	+++	4.1	Y	+ wk

Y = Lesions present consistent with PPR infection.

N = No significant histopathological findings.

NS− No sample.

IHC = Immunohistochemistry score: +wk = weak immunostaining (<20 cells);+ = mild immunostaining (<25% of the section);++ = moderate immunostaining (25% to 50% of the section);+++ = abundant immunostaining (51% to 75% of the section);++++ = extensive immunostaining (>75% of the section).

### Quantification by Real-time RT-PCR

RNA from tissue homogenates, as well as oral/nasal swabs, was extracted using the RNeasy Mini kit (Qiagen, USA) while RNA from whole blood was extracted using the QiaAmpRNA blood Mini kit (Qiagen, USA) as described by the manufacturer. Quantification by real-time RT-PCR was performed using primers specific to PPRV that were designed using Genscript software (USA). The expected 130 bp fragment, covering part of the PPRV N protein gene, was previously described by Saravanan *et al.* [28] (Genbank #DQ840168): NrF1 5′TGACCAGGGAAGAAGTCACA 3′, NrR1 5′TCGTCTTCAGGCATGATCTC 3′ and NrP 5′Fam TTGTCCTTCTCGTCGGGCCC 3′Tam. The real-time RT-PCR reaction was performed using an ABI 7500 Sequence Detection System (Applied Biosystems, USA) and a protocol previously described by Bao *et al*. [29]. The reaction mixture contained 5 µl of extracted RNA, 12.5 µl of 2× Quantitect Probe master mix (Qiagen), 0.25 µl Quantitect Enzyme, 10 µM of forward and reverse primers (1 µl each) and 10 µM of TaqMan probe (1 µl) and 17.75 µl of water for a final volume of 25 µl for each sample analyzed. The following thermal profile was used: an initial reverse transcription step at 50°C for 30 minutes, followed by 95°C for 15 minutes and 45 cycles of amplification (15 s at 94°C and 1 minute at 60°C). The data generated was then analyzed using the SDS 1.2 software program (Applied Biosystems, USA).

### Peste Des Petits Ruminants Antibody ELISA

Peste des petits ruminants viral antigen was purified from virus amplified using Vero cells incubated for 17 days. The viral suspension was layered onto a PBS (pH 7.4)/20% sucrose gradient and pelleted by ultracentrifugation at 118,000×g for 2 hours. The pellet was then resuspended in PBS (pH 7.4) and stored at −80°C for use as antigen for the indirect ELISA. Ninety-six well ELISA plates (Nunc, USA) were coated with purified virus (diluted 1∶400 in carbonate buffer [pH 9.6]) and incubated overnight at 4°C. The plates were then incubated with blocking solution (5% skim milk in PBS/0.05% Tween) for 1 hour at 37°C. Serially diluted sheep and goat sera (starting at a 1∶50 dilution) were then added, incubated for 1 hour at 37°C, washed 3 times, and further incubated for 1 hour at 37°C with a 1∶1000 dilution of alkaline phosphatase-conjugated donkey secondary antibody (Rockland, USA). The plates were then washed 3 times, developed using Blu Phos™ phosphatase substrate (KPL, USA) and absorbance measured at a wavelength of 650 nm. Endpoint titres were determined using the average optical density plus two standard deviations from 300 negative sheep sera as the cut-off value.

### Virus Neutralization Test (VNT)

Virus neutralizing antibodies were measured using VNT. Serial dilutions (from 1∶20 to 1∶20,480) of sheep and goat sera, starting from 4 to 21 dpi, were evaluated. PPRV (100 TCID_50_, in DMEM) was mixed with sheep and goat sera (in duplicate) to a volume of 200 µl, and incubated for 1 hour at 37°C. Vero cells incubated in 96-well plates were infected with the 200 µl mixture of virus and serum (or media, negative control). The cells were then incubated for 15 to 18 days, with daily examination for CPE.

### ELISA-based Detection of Sheep and Goat Interferon-gamma (IFN-γ)

A bovine quantitative IFN-γ kit (AbD Serotec, USA) was used to measure sheep and goat IFN-γ based on the ability of selected bovine monoclonal antibodies to cross-react with both sheep and goat IFN-γ. Serum IFN-γ levels were measured by quantitative ELISA, as previously described by Tourais-Esteves *et al.* [30]. Total IFN-γ concentrations were determined by normalization with a standard provided by the manufacturer.

### Statistics

Statistics were performed using a t-test with Excel (Microsoft) to determine differences between sheep and goats for viral RNA loads, serology and IFN-γ levels in serum.

## Results

### Clinical Disease Progression

**Figure 1:**
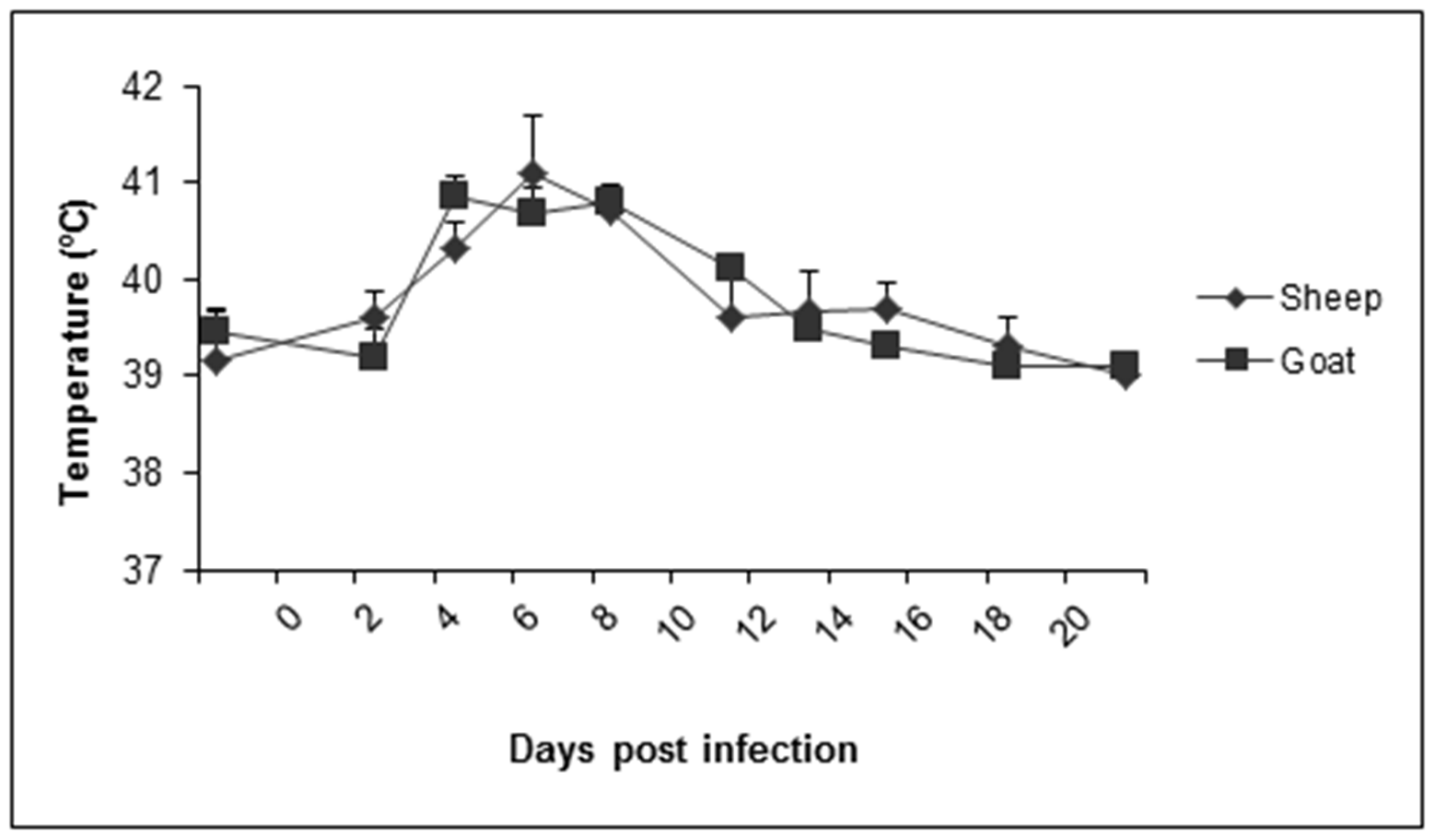
Rectal temperatures of sheep and goats following PPRV infection. Rectal temperatures of sheep and goats were measured 2 days prior to experimental infection with PPRV (Malig strain), and following infection at regular intervals until 21 dpi. Results presented are the mean temperatures with standard deviations from animals at each time point.

Sheep and goats were allowed to acclimate to the laboratory environment for a period of two weeks prior to experimental infection with PPRV. During that time, all experimental animals were healthy and free of disease, with normal rectal temperatures ranging from 39.5–40.3°C (sheep) and 39.1–40.1°C (goats). Following inoculation with PPRV, clinical signs, including mild depression, moderate bilateral mucopurulent nasal discharge and elevated rectal temperatures (40.5–41.1°C), were observed in goats starting at 4 dpi (Figure 1). In sheep, a similar increase in temperature was also observed at 4 dpi, however, no other clinical signs of disease were observed at this stage.

**Figure 2:**
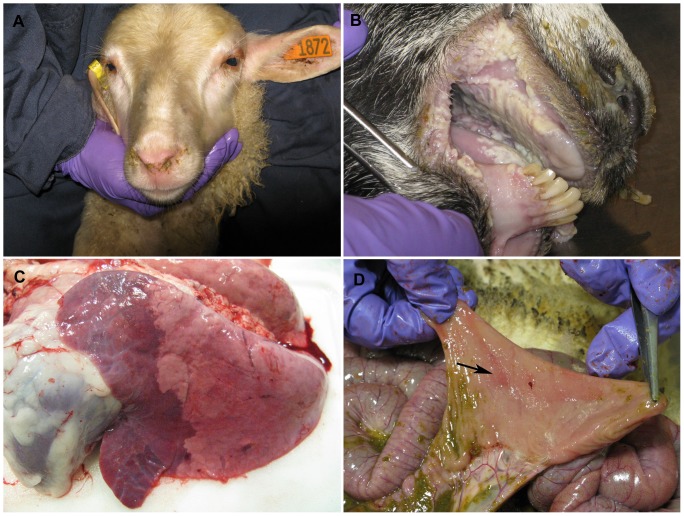
Clinical signs and gross pathology in sheep and goats following infection with PPRV (Malig strain). (A) Nasal discharges were observed in sheep at 6 dpi. (B) At 8 dpi, goats developed significant erosions of oral mucosa, as well as (C) bronchointerstitial pneumonia. (D) Depressed and reddened Peyer’s patches from infected sheep at 11 dpi.

The progression of the disease was most pronounced between 6 and 8 dpi in both sheep and goats, where significant inactivity and nasal discharges were observed in nearly all animals (Figure 2A). Furthermore, rectal temperatures were at their highest during those periods, with measurements in goats ranging from 40.3–41.6°C (8 dpi) and sheep from 40.8–42.3°C (6 dpi). Following these time points, both groups of animals steadily recovered from all clinical signs, with rectal temperatures returning to normal at 13 dpi. By 18 dpi, no clinical signs were observed among all remaining sheep and goats.

### Gross Pathology of PPRV Infection in Sheep and Goats

In the one sheep sampled at 6 dpi, small oral ulcers (2–5 mm) were observed as well as diffuse pulmonary edema and bronchopneumonia. Mesenteric lymph nodes were enlarged and pinpoint areas of erosion were evident in the Peyer’s patches. In the goat sampled at 6 dpi, only mild enlargement of the prescapular and mesenteric lymph nodes was observed.

At 8 dpi, in the single sheep and goat sampled there were enlarged mesenteric lymph nodes. The sheep had milder lesions, including 1–2 mm erosions of the oral mucosa and multifocal erosions (1.0 to 1.5 cm) throughout the ileum. The goat sampled at 8 dpi showed more severe lesions at this time point including: conjunctivitis, widespread and severe necrosis and erosion of oral mucosa (Figure 2B), small erosions in larynx and esophagus, bronchointerstitial pneumonia (Figure 2C), cecal/colonic hemorrhage/necrosis and enlargement of prescapular and parotid lymph nodes.

In the single sheep sampled at 11 dpi, there were mild lesions including enlarged mesenteric lymph nodes and depressed and reddened Peyer’s patches (Figure 2D). In the single goat sampled at 11 dpi, there was conjunctivitis, mild enlargement of the parotid lymph nodes and bronchointerstitial pneumonia.

### Immunohistochemistry (IHC), Histopathology and Viral RNA Quantification in Goat and Sheep Organs

Following PPRV inoculation, a sheep and goat were euthanized at each of 6, 8 and 11 dpi and tissue samples were collected from multiple organs. Tissue tropism for PPRV was determined using a combination of histology, immunohistochemistry and real-time RT-PCR. Tables 1 and 2 summarize the results for both sheep and goats, respectively. In general, though the lesions observed in both the sheep and goats were similar, they varied in severity. The most severe lesions were observed in the single sheep euthanized at 6 dpi and the single goat euthanized at 8 dpi. In both species, lesion severity decreased in most tissues by 11 dpi. Abundant viral antigen was detected by immunohistochemistry at 6 and 8 dpi for most organs sampled in both species, however at 11 dpi the immunostaining was weak and only observed in a few cells.

In the goat sampled at 6 dpi, high levels of antigen were observed in lymphoid organs including the tonsil, spleen and parotid/bronchial lymph nodes, with some involvement of the intestine. In the goat sampled at 8 dpi, detection of virus by both IHC and real-time RT-PCR was highest in the lungs, intestines and oral mucosa. In contrast, high viral loads were detected at in the sheep sampled at 6 dpi in the lymphoid tissues, as well as the respiratory and intestinal tracts of sheep. It should also be noted that at 6 and 8 dpi considerable virus was detectable in the spleen and tonsils of both animal species. In all cases, the level of detectable virus diminished considerably at 11 dpi. When the presence of PPRV was quantified using real-time RT-PCR, the amount of viral RNA was generally consistent with the results from IHC, confirming viral replication.

**Figure 3:**
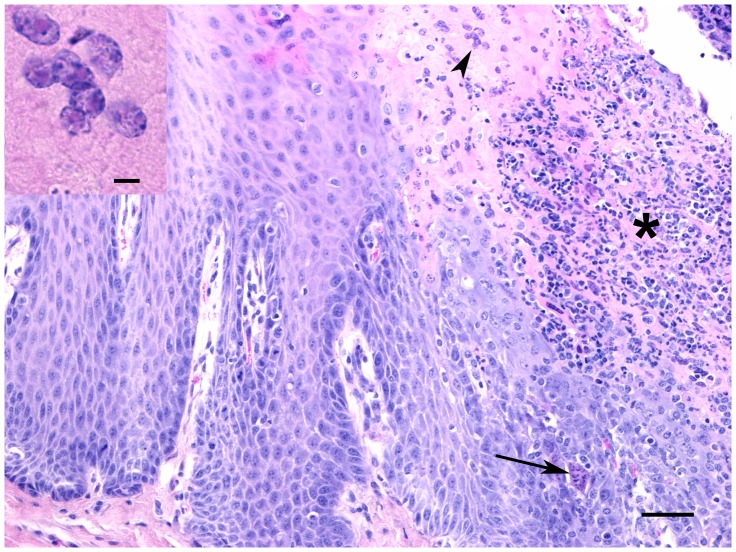
Cross-section through a tonsil from a goat at 8 dpi. There is necrosis of surface epithelium and extensive neutrophilic infiltrate (*) as well as occasional syncytial cells (arrow) and intranuclear inclusion bodies in upper epithelial layers (arrowhead, see inset). HE stain, bar = 50 µm. Inset: Eosinophilic intranuclear inclusion bodies. Bar = 5 µm.

**Figure 4:**
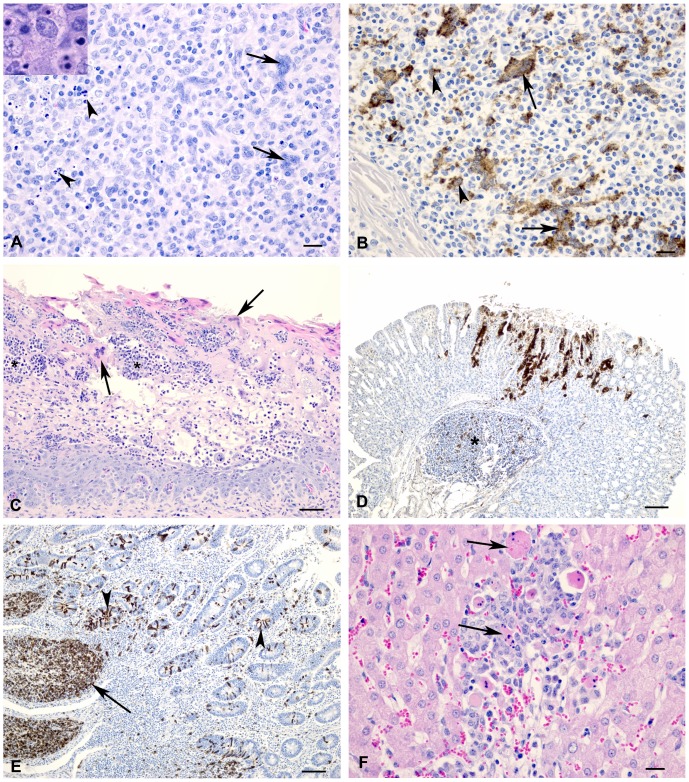
Histology and immunohistochemistry of sheep and goat tissue at varying time points following infection with PPRV (Malig strain). (A) Section of a lymph node; goat, 6 dpi. Multinucleated syncytial cells (arrows) and degenerating or apoptotic lymphocytes (arrowheads) were observed at 6 and 8 dpi. Inset: Higher magnification showing detail of apoptotic lymphocytes. HE stain, bar = 20 µm (B) Lymph node; goat, 6 dpi. Positive immunostaining using PPRV-specific antibodies in syncytial cells (arrows) and macrophages (arrowheads). Bar = 20 µm. (C) Section of omasum; sheep, 8 dpi. There is necrosis and loss of epithelium with edema, neutrophil infiltration (*) and syncytial cell formation (arrows). HE stain, bar = 50 µm. (D) Abomasum; goat, 8 dpi. Positive immunostaining for PPRV antigen could be detected within the gastric pits and glands as well as in the associated lymphoid tissue (*). Bar = 100 µm. (E) Ileum; sheep, 6 dpi. There is positive immunostaining for PPRV antigen within Peyer’s patches (arrow) as well as crypt epithelial cells (arrowhead). Bar = 50 µm. (F) Liver; sheep, 8 dpi. Focal area of hepatocyte loss with non-suppurative inflammation and degenerating syncytial cells (arrows). HE stain, bar = 50 µm.

In both species at 6 and 8 dpi, there were prominent lesions in the palatine tonsils, which included necrosis of surface and crypt epithelium with infiltration of neutrophils, formation of syncytial cells and scattered intranuclear inclusion bodies (Figure 3). Lymph node lesions were characterized by lymphocyte depletion (primarily in the cortical lymphatic nodules) and there were numerous lymphocytes with pyknotic or karyorrhexic nuclei. Throughout the nodes there were numerous multinucleated syncytial cells and apoptotic cells (Figure 4A). At 11 dpi, cortices were often thin and lymphoid nodules were not prominent, and in some cases there was hyperplasia of the paracortex. The lymphoid tissue of both the palatine tonsil and third eyelid appeared similar to the lymph nodes, with lymphocyte depletion and presence of syncytial cells. In the spleen, the white pulp areas were depleted of lymphocytes and the red pulp appeared hypercellular. Splenic syncytial cells were only observed in the sheep. Using IHC on the lymphoid tissues, antigen was frequently detected in macrophages and syncycial cells (Figure 4B), as well as in dendritic reticular cells and occasional lymphocytes.

Severe and widespread microscopic lesions in the pharyngeal, oral and nasal mucosa were only observed in the goat examined at 8 dpi. In the sheep at 6 and 8 dpi and the goat at 6 dpi, these lesions were smaller and only rarely observed. Lesions were characterized by multifocal erosions and formation of syncytial cells in upper cell layers, and necrosis and loss of epithelium with replacement by edema fluid and neutrophils. In the forestomachs of a few animals, multifocal areas of epithelial necrosis, with neutrophil infiltration and syncytial cell formation, were observed (Figure 4C). Positive immunostaining was observed within epithelial cells. Abomasal necrosis was only observed in the goat at 8 dpi and antigen could be detected multifocally within the gastric pits and glands (Figure 4D). Intestinal lesions were most severe at 6 and 8 dpi. In both species, lesions were observed within the duodenum, jejunum and ileum, with the ileum showing the most severe changes. Lesions were characterized by blunted villi, degeneration of surface and crypt epithelial cells, expansion of lamina propria by a primarily mononuclear infiltration with scattered syncytial cells and severe depletion of lymphocytes within Peyer’s patches. Significant lesions, consisting of lamina proprial inflammation with scattered syncytial cells and multifocal crypt necrosis, were observed in both species, however, in the single goat at 8 dpi, diffuse necrosis and inflammation was observed. Positive immunostaining was observed extensively within Peyer’s patches, as well as within surface and crypt epithelial cells, syncytial cells and inflammatory cells within the lamina propria (Figure 4E). In the liver at 8 dpi, there were multifocal areas of hepatocyte loss with non-suppurative inflammation and formation of syncytial cells (Figure 4F).

**Figure 5:**
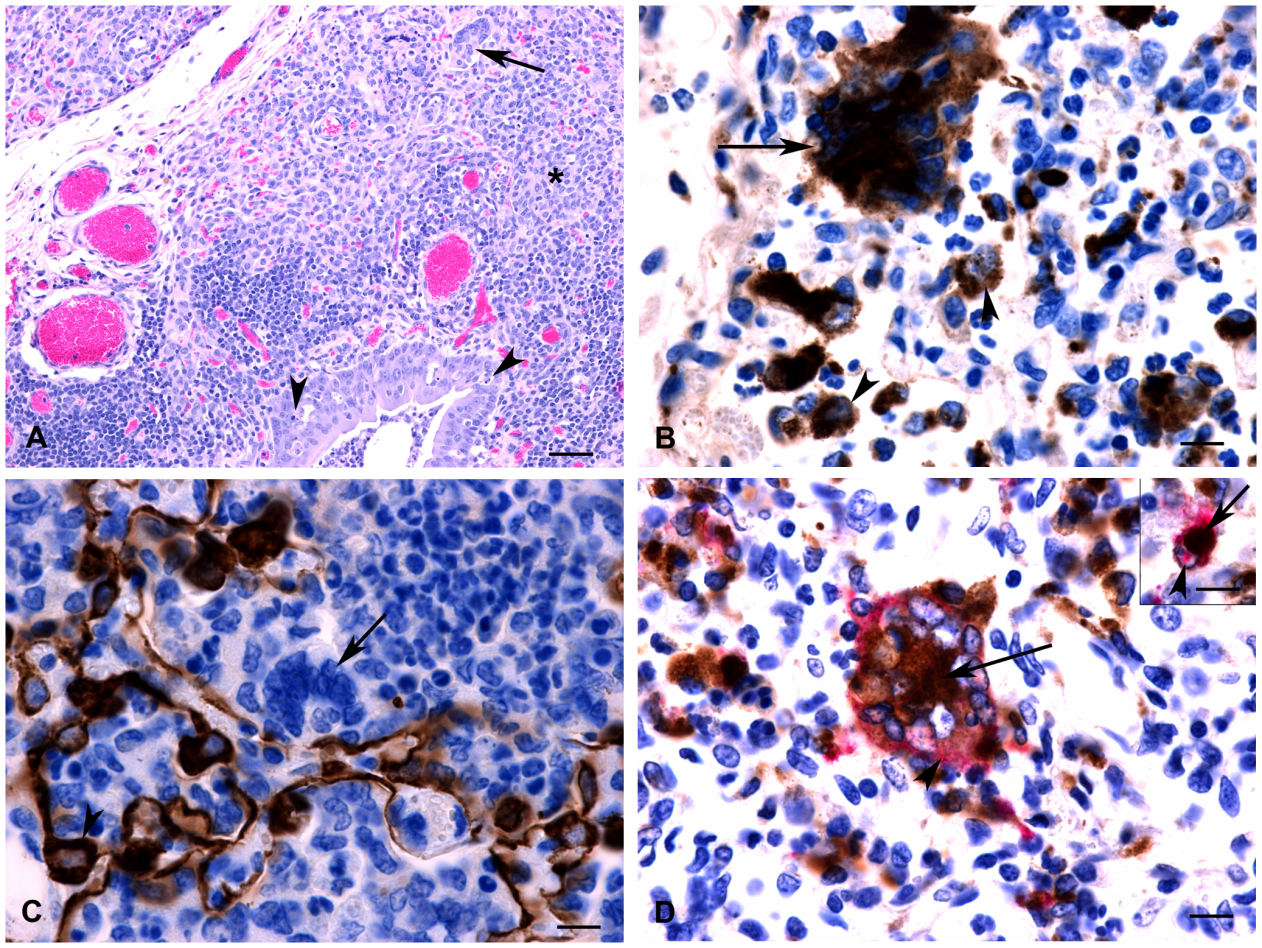
Histopathology and immunohistochemistry of goat lung at 8 dpi. (A) Hyperplasia of bronchiolar epithelium is evident with scattered epithelial degeneration (arrowheads) and abundant neutrophils within the lumen. Surrounding parenchyma is consolidated (*) with severe infiltration of mononuclear inflammatory cells. Note large syncytial cell (arrow). (B) Positive immunolabelling for CD68 was observed within multinucleated syncytial cells indicating they are of monocyte/macrophage origin (arrow). Note positive immunostaining of adjacent macrophages (arrowheads). (C) Positive immunolabeling for cytokeratin is observed within pneumocytes (arrowhead); however, syncytial cells are negative (arrow) indicating that they are not of epithelial origin. (D) Double immunolabelling detected the simultaneous expression of CD68 macrophage marker (brown stain, arrow) and PPRV antigen (pink stain, arrowhead) within multinucleated syncytial cells. Inset: Double immunolabelling detected expression of CD68 macrophage marker (brown stain, arrowhead) and PPRV antigen (pink stain, arrow) within the same cell indicating the presence of viral antigen within macrophages. Bar = 10 µm.

At 6 dpi in the single sheep sampled and at 8 dpi in the single goat sampled, severe bronchointerstitial pneumonia was observed in the cranial and middle lung lobes (Figure 5A). There was multifocal suppurative and necrotizing bronchiolitis, with variable epithelial attenuation to hyperplasia and occasional intracytoplasmic inclusion bodies. The alveolar walls were expanded by inflammatory cells and hyperplastic type II pneumocytes. There was multifocal consolidation with infiltrates of mixed inflammatory cells. Many of the infiltrating inflammatory cells could be definitively identified as macrophages when CD68 immunolabelling was performed (Figure 5B), although neutrophils and lymphocytes were also observed. Syncytial cells were observed within alveolar spaces and bronchiolar-associated lymphoid tissue (BALT), showing positive immunostaining for CD68, but negative for cytokeratin immunostaining, indicating that the syncytial cells were of macrophage origin (Figure 5B and 5C). Positive immunostaining for viral antigen was observed in bronchial/bronchiolar epithelium, in cells morphologically identified to be alveolar macrophages, syncytial cells and macrophages/lymphocytes within the BALT. Double-immunolabeling was performed to confirm that both syncytial cells and macrophages were infected (Figure 5D). Lung lesions were similar, but, milder in the other animals examined, with the exception of the goat sampled at 8 dpi, in which there was also a severe fibrinosuppurative bronchopneumonia suggestive of secondary bacterial infection.

### Virus Isolation

In order to confirm that PPRV was replicating in sheep and goats, virus isolation was attempted on samples collected from various tissues. In sheep, virus was successfully isolated from third eyelid tissue at 6 dpi. In goats, virus was isolated from skin/lip tissue and conjunctiva at dpi 6, oral mucosa on 8 dpi and skin/lip tissue on 11 dpi. All isolations were confirmed to be PPRV by sequencing of the N gene. Virus isolation was unsuccessful in all other organs tested, including lymph nodes.

### Quantification of PPRV Using Real-time RT-PCR in Whole Blood, Oral and Nasal Swabs

**Figure 6:**
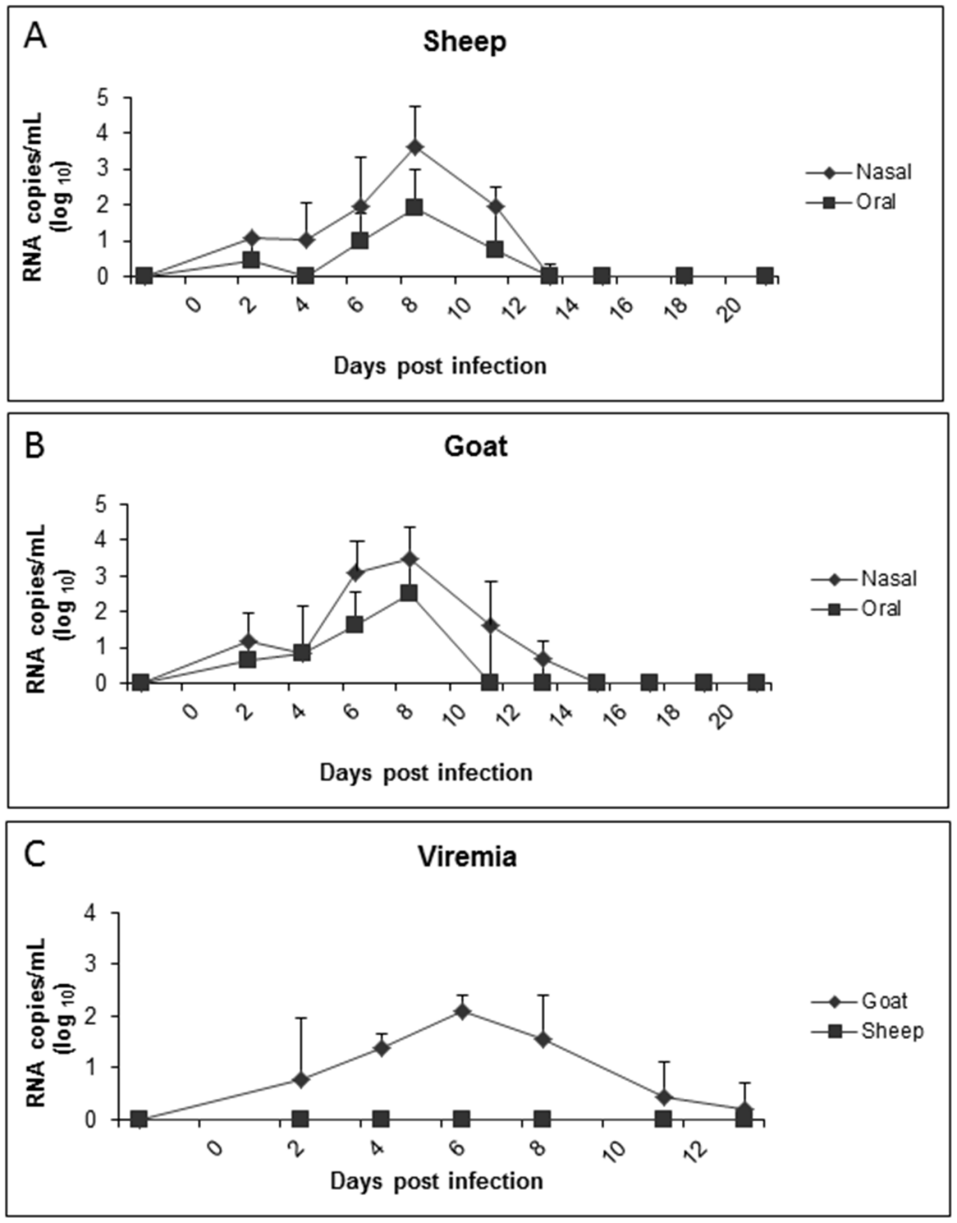
Quantification of PPR viral RNA in blood, nasal and oral swabs determined using real-time RT-PCR. Both nasal and oral swabs were collected from sheep (A) and goats (B) at various time points until 21 dpi. Viral RNA quantification from whole blood (C) was also performed at identical time points. Note that in the case of sheep (C), viral RNA was not detectable at any time point before or after experimental infection with PPRV. Results presented are the mean value with standard deviation from animals at each time point. P<0.05 for sheep and goat nasal swabs at 6, 8 and 11 dpi and for sheep and goat oral swabs at 6 and 8 dpi compared to −2 dpi by t-test. P<0.05 for goats whole blood at 4, 6 and 8 dpi compared to −2 dpi by t-test.

In order to detect the presence of PPRV RNA at mucosal surfaces of sheep and goats, oral and nasal swabs were collected at various time points following experimental infection. Viral RNA was quantified using real-time RT-PCR. In both sheep and goats, PPRV was detectable as early as 2 dpi in oral swabs with 1/6 goats and 2/6 sheep as well as nasal swabs with 3/6 goats and 4/6 sheep showing detectable levels of viral RNA (Figure 6). Significant detection of PPRV RNA was observed in nasal swabs from sheep and goats on 6, 8 and 11 dpi and in oral swabs from sheep and goats at 6 and 8 dpi. There were no significant differences in viral RNA loads between sheep and goats at any time point. In all cases, the highest viral RNA loads were detected at 8 dpi, for both oral and nasal swabs. In both sheep and goats, viral RNA shedding decreased by 13 dpi, with none of the remaining sheep and only 2 of the remaining goats having detectable levels of viral RNA in nasal swabs. All remaining sheep and goats had no detectable viral RNA in nasal swabs past 13 dpi, remaining negative until the end of the study.

To measure viremia, whole blood was assessed for PPRV RNA using quantitative real-time RT-PCR. Low levels of viral RNA were detected in whole goat blood at 2 dpi in 2 goats. Significant levels of PPRV RNA were detected in goats at 4, 6 and 8 dpi, with copy numbers never exceeding 1000 copies/mL. This is in contrast to sheep, where PPRV RNA was not detected in whole blood at any time point post infection.

### Generation of PPRV-specific Antibodies in Response to Viral Infection

**Figure 7:**
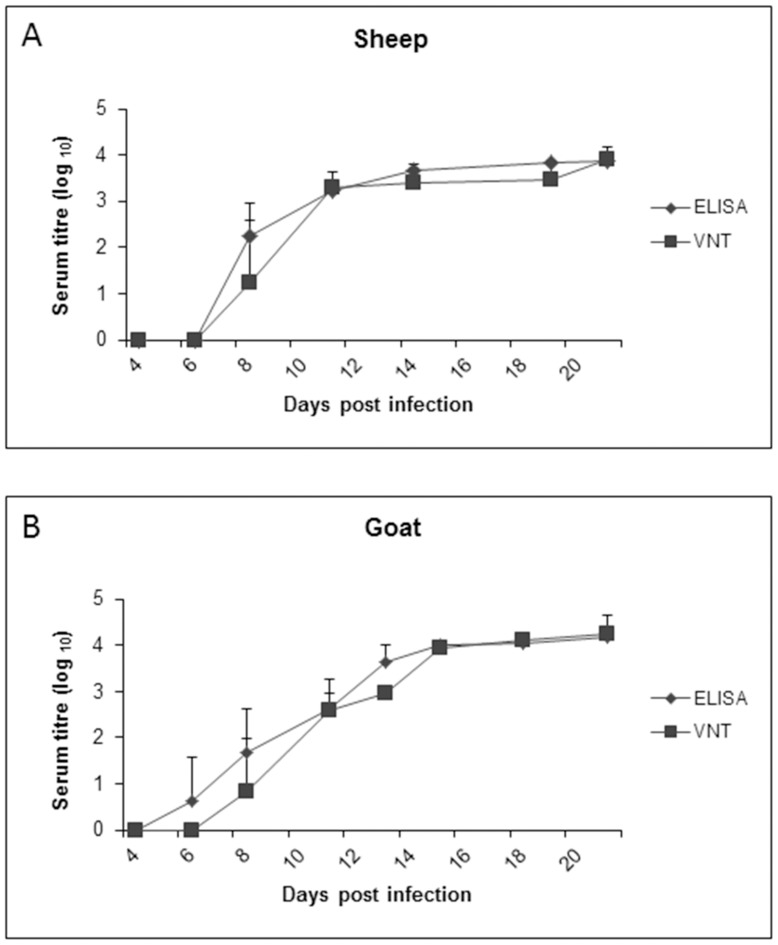
Seroconversion following experimental PPRV infection. PPRV-specific antibody titres in serum from sheep (A) and goats (B) measured using an indirect ELISA and virus neutralization test (VNT). Results presented are the mean values with standard deviations from animals at each time point. P<0.05 for ELISA from sheep and goats starting at 8 dpi compared to −2 dpi and P<0.05 for VNT from sheep and goats starting at 11 dpi compared to −2 dpi by t-test.

In order to quantify PPRV-specific IgG antibodies generated in sheep and goats following viral infection, sera were collected at regular time intervals between 4 and 21 dpi. PPRV-specific antibodies were measured in serum using an indirect PPRV ELISA. Seroconversion for both sheep and goats started at 8 dpi as measured by the indirect ELISA (Figure 7). Neutralizing antibody levels against PPRV were measured using VNT. Similar kinetics of sero-conversion to the ELISA was observed with the VNT in sheep and goats, with significant neutralizing antibodies elicited starting at 11 dpi. The antibody titers determined by ELISA and VNT remained at significant levels in all animals until the termination of the study at 21 dpi and there were no significant differences between the antibody responses in sheep and goats.

### Quantification of IFN-γ in Sheep and Goat Sera Following PPRV Inoculation

**Figure 8:**
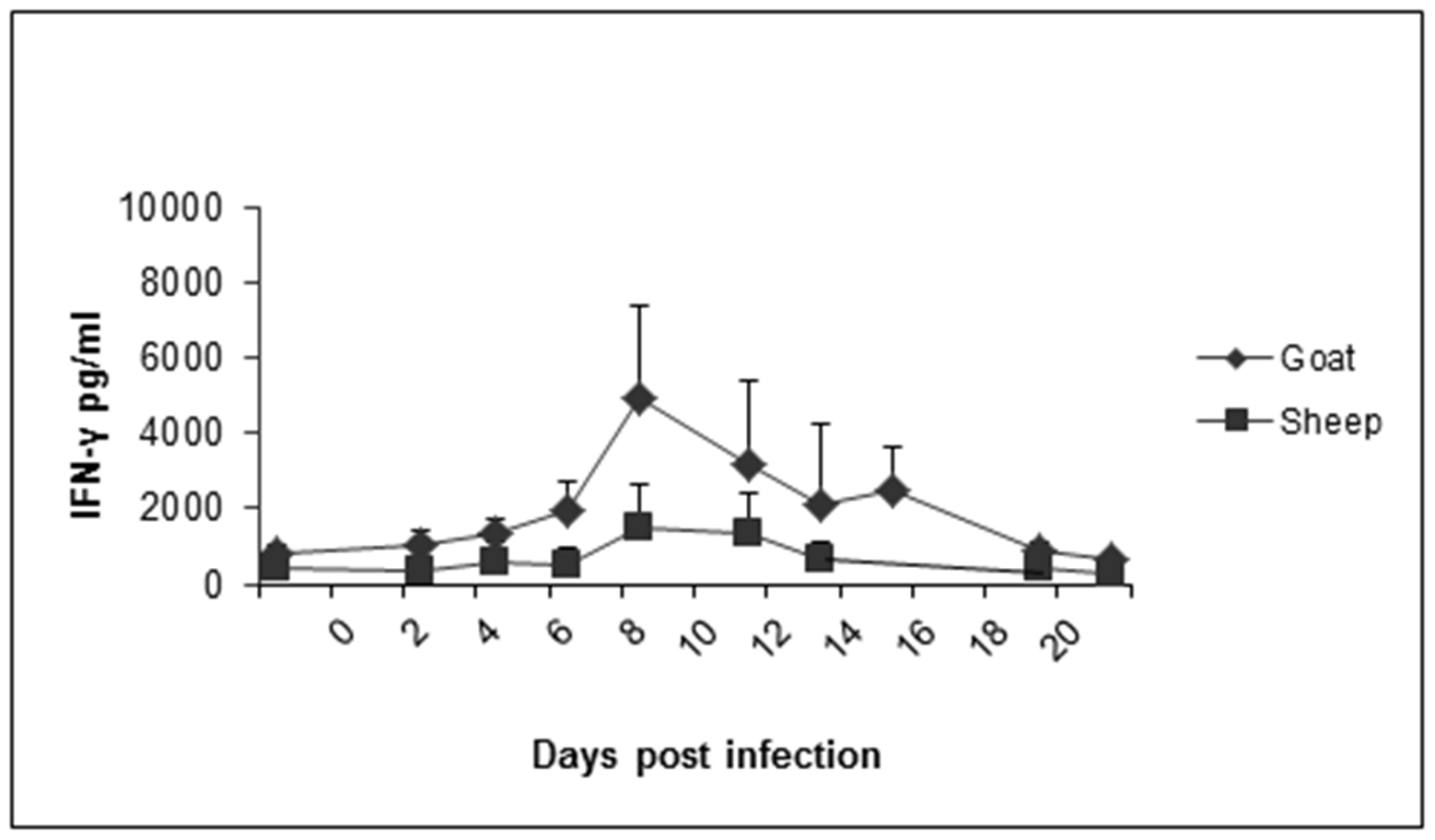
Quantification of interferon-gamma (IFN-γ) in serum samples collected from sheep and goats following PPRV infection. Measurement of IFN-γ levels were performed using an IFN-γ ELISA and cross-reactive antibodies against bovine IFN-γ, and quantified using a standard provided by the manufacturer. Results presented are the mean values with standard deviations from animals at each time point. P<0.05 for goats at 8 dpi compared to −2 dpi by t-test.

Serum samples obtained following PPRV inoculation were assayed and quantified for the presence of IFN-γ in response to PPRV infection. No significant IFN- γ was detected in sheep at any timepoint. Goats showed a significant increase in IFN-γ on 8 dpi compared to samples at −2 dpi (Figure 8).

## Discussion

Over the past three decades, multiple studies on PPR have been performed in experimental settings [10]–[13], [15], [16], [31] and although sheep and goats have been used in models for experimental infection, few studies have ever utilised both sheep and goats in parallel. This is of particular importance, since data suggesting that goats are more susceptible to PPRV infection than sheep are based on epidemiologic studies described by Taylor nearly 30 years ago [10]; however, the difference in susceptibility between the two species has never been thoroughly investigated. When compared, viral RNA loads in tissues for both species were similar, as well as viral RNA levels from nasal and oral swabs in both sheep and goats peaked at day 8 following infection. The difference in viral replication was limited to viral RNA detected in whole blood where goats had detectable levels of PPRV RNA, as opposed to the absence of measurable viral RNA in whole sheep blood.

Lesions and viral antigen were primarily observed in the respiratory and gastrointestinal tract, as well as within lymphoid organs, which is in agreement with previous studies [13], [16]. An interesting feature was the observation of syncytial cells within most of the affected tissues, including lymph nodes, lung, spleen, tonsil, liver, oral/nasal epithelium, rumen, omasum, intestines and third eyelid. While the formation of syncytia is a common feature of morbillivirus infection, in previous experimental PPRV infection studies the presence of syncytial cells within these tissues has been variable [13], [16], [32]. Immunostaining using the macrophage marker CD68 revealed that syncytial cells in the lungs of PPRV-infected animals are of monocyte/macrophage lineage, suggesting they are derived from alveolar macrophages. This is in contrast to other morbillivirus infections such as measles, in which the syncytial cells in the lungs have been described as arising from epithelium [33]. In addition, double-immunolabeling revealed these syncytial cells to contain abundant PPR viral antigen. Furthermore, a large proportion of the infiltrating inflammatory cells were determined to be of macrophage origin and many of these also contained viral antigen. This suggests that the alveolar macrophages may play a significant role in the pathogenesis associated with PPRV infection, although this may depend on the route of infection. In recent studies with aerosol infection of measles virus, it has been shown that the virus enters the host by infection of alveolar macrophages and/or dendritic cells in the airways, and is amplified in local lymphoid tissues [34].

While both sheep and goats had similar patterns of gross pathology, following nearly identical time courses, PPRV-induced pathology was significantly more pronounced in the single goat sampled at 8 dpi with widespread and severe oral lesions, as well as significant haemorrhaging and necrosis of cecal and colonic tissue, being observed. There were also noticeable differences in the degree of enlargement of lymph nodes between the two species, with goats being more severe. The degree to which secondary lymphatic organs, as well as mucosal and gut-associated lymphatic tissue, are affected is consistent with previous studies of morbillivirus tropism [35]. Viral loads in the intestine were high based on real-time RT-PCR and IHC in both sheep and goats. Although we did not see gross lesions in the sheep, both species showed similar histopathological lesions with severe depletion of Peyer’s patches.

The viremia in goats elicited a more robust inflammatory response, as indicated by increased IFN-γ levels observed in goat sera compared to sheep. These results appear to be in agreement with previous work using a highly virulent Indian strain of PPRV [13]. Antibody responses, including neutralizing antibodies, were similar between sheep and goats.

In previous studies investigators administered PPRV either intranasally, subcutaneously or using both routes, as in this study. The degree of clinical progression appeared to follow a time course similar to that described in previous experiments, thereby suggesting that the route of administration did not change disease progression when compared to that of earlier studies. Recent work by Pope *et al.* [16], and El Harrak *et al.* [15] showed that the severity of clinical signs of infected goats peaked between 6 and 8 dpi. These results are consistent with the observations of this study, even though a different strain of PPRV was used. In this study, a passaged PPRV strain (Malig), previously isolated from a PPR outbreak in Yemen [12], was used for both sheep and goat infections. Partial sequencing of this strain suggests a high degree of homology with the more well-characterized attenuated Nigeria 75/1 strain (data not shown), phylogenetically classified as a Group I lineage of the virus [9]. This is in contrast to more recent studies, where either the Cote d’Ivoire (Group II) or a Moroccan field isolate (unknown classification), were used. Despite using different virus isolates, the clinical signs observed in the previous experimental studies indicate that the course of disease is highly conserved between phylogenetically distant lineages of PPRV. However, it should be noted that the virulent Indian isolate, Izatnagar/94, a Group IV lineage isolate, did induce between 80–90% mortality in experimentally infected goats [13].

The histopathology, IHC, and quantitative RT-PCR results from both sheep and goats also provide some insight into the disease progression of PPR in small ruminants. In both species, significant levels of virus were detected in the lymph nodes, lymphoid tissues and digestive tract at 6 dpi. However, within two days thereafter, viral loads were lower in most lymph nodes, but the presence of virus increased in the tissues of the digestive tract in both sheep and goats. These results, when combined with the gross pathology data for both species, suggest that primary replication of PPRV may occur in the draining lymph nodes, which then seed the organs of the digestive and respiratory tract.

Both sheep and goats developed clinical signs of PPR, although sheep did not have detectable viral RNA in blood, compared to goats. The reason for this difference in viral replication is not known, but demonstrates that although PPRV can infect both sheep and goats, there are differences depending on the host. A similar situation is observed with capripoxvirus where there are differences in the susceptibility of sheep and goats, although the differences are much more pronounced depending on the virus isolate involved [36].

An important discrepancy between the experimental model developed in this study (as well as most previous studies) and field conditions pertains to the lack of mortality in both sheep and goats in experimental settings, compared to the high degree of PPR-related deaths of livestock observed following field outbreaks. As mentioned in previous publications, this difference may be due to: 1) differences in the breed of sheep or goat used in the studies; 2) the overall health of the animals; 3) the virus strain/isolate used and how it was amplified or 4) the absence of other bacterial, viral or helminth pathogens, which may compromise the host immune responses. For example, when PPRV was co-administered with the bacterial pathogen *Mannheimia haemolytica*, enhanced pneumonia was observed [37]. In addition, in natural field settings livestock are likely to be co-infected with other viruses such as capripoxvirus and/or bluetongue virus [38], that would most likely exacerbate the onset and severity of PPR.

The experimental PPRV-infection model developed and described in this paper in sheep and goats will now be used as a standard model, allowing for more in-depth pathogenesis studies of PPRV infection in the future as well as the evaluation of PPRV vaccines. Despite the complete recovery of experimentally infected animals, both sheep and goats developed clinical signs of disease, had detectable levels of virus replication and developed PPRV-specific antibodies.

Since the 1970’s, when it was found that attenuated rinderpest virus could confer protection against PPRV, experimental vaccines based on attenuated virus, as well as recombinant viruses, have been developed. These include the attenuated Nigerian 75/1 PPRV strain, as well as the south Asian strains, Sungri 96, Arasur 87, and Coimbatoire 97 [20]. Furthermore, recombinant viruses expressing either PPRV H- and/or F-protein have been demonstrated as potential recombinant vaccines using capripoxvirus, vaccinia virus or adenovirus as vectors [22], [23], [25], [26], [39], [40]. As novel vaccine candidates continue to be developed [41], the need to evaluate these vaccines in both sheep and goats arises. The understanding of the pathogenesis and the development of a reproducible PPR infection model in both sheep and goats will allow this to occur.
